# The Anterior Cingulate Cortex Promotes Long-Term Auditory Cortical Responses through an Indirect Pathway via the Rhinal Cortex in Mice

**DOI:** 10.1523/JNEUROSCI.2252-22.2023

**Published:** 2023-06-07

**Authors:** Ye Liang, Jing Li, Yu Tian, Peng Tang, Chunhua Liu, Xi Chen

**Affiliations:** ^1^Department of Neuroscience, City University of Hong Kong, Kowloon Tong, Hong Kong SAR, 0000, P.R. China; ^2^Centre for Regenerative Medicine and Health, Hong Kong Institute of Science & Innovation, Chinese Academy of Sciences, Hong Kong SAR, 0000, P.R. China; ^3^City University of Hong Kong Shenzhen Research Institute, Shenzhen, 518057, China; ^4^Key Laboratory of Biological Targeting Diagnosis, Therapy and Rehabilitation of Guangdong Higher Education Institutes, The Fifth Affiliated Hospital of Guangzhou Medical University, Guangzhou, 510799, China

**Keywords:** anterior cingulate cortex, auditory cortex, long-term enhancement, rhinal cortex, top-down modulation

## Abstract

Sensory cortical areas are robustly modulated by higher-order cortices. Our previous study shows that the anterior cingulate cortex (ACC) can immediately and transiently enhance responses in the mouse auditory cortex (ACx). Here, we further examined whether strong activation of ACC neurons can induce long-term effects in mice of both sexes. To our surprise, only stimulation of cell bodies in the ACC, but not ACC-to-ACx terminal activation, induced long-term enhancement of auditory responses in the ACx. Anatomical examination showed that the ACC indirectly projects to the ACx via the rhinal cortex (RCx). High-frequency stimulation of ACC-projecting terminals to the RCx or RCx-projecting terminals to the ACx induced a similar effect as the cell body activation of ACC neurons, whereas silencing the RCx blocked this long-term enhancement. High-frequency stimulation of ACC projections to the RCx also induced long-term augmentation of sound-evoked flight behavior in male mice. These results show that the ACC promotes the long-term enhancement of auditory responses in the ACx through an indirect pathway via the RCx.

**SIGNIFICANCE STATEMENT** In this study, we demonstrate that the anterior part of the anterior cingulate cortex (ACC) evokes long-term enhancement of auditory responses in the auditory cortex (ACx) when it is strongly activated. Importantly, instead of a direct projection, we show that the ACC implements this effect via an indirect pathway through the lateral rhinal cortex using a series of physiological, optogenetic, anatomic, and behavioral experiments. Along with a short-term effect, this long-term enhancement induced by an indirect ACC-to-ACx projection could increase the odds of survival when animals are faced with threats after a significant event.

## Introduction

Primary sensory cortical areas (e.g., auditory cortex [ACx]) are adaptive processors that are subject to robust top-down modulation by high-order brain areas. These areas are also strongly influenced by various statuses of the organisms, such as arousal, attention, and perceptual learning. Regions of the prefrontal lobe that send top-down projections to the sensory cortex are known to play a critical role in high-level cognitive functions, such as attentional control and working memory (D. Liu et al., 2014; Kamigaki and Dan, 2017; Nabel et al., 2020; P. Y. Wang et al., 2020; Y. Liu et al., 2021; Y. Zhang et al., 2022). In particular, the anterior cingulate cortex (ACC), located in the dorsal frontal cortex, is tightly involved in the integration of advanced cognitive and emotional functions (Pardo et al., 1990; Bush et al., 2000; Hughes and Beer, 2012; Akam et al., 2021; Kim et al., 2021). Our recent work shows that direct activation of anterior ACC (aACC)-to-ACx projections enhances responses to acoustic noise stimuli in the ACx and immediately facilitates sound-evoked flight behavior (within tens of milliseconds) (Sun et al., 2022). However, this facilitating effect has only been examined on a short-term scale (within 1 s) but not a long-term one. If long-term enhancement of auditory responses in the ACx can be induced by ACC activation, the next question would be how the ACC induces this long-lasting effect.

Therefore, we first examined whether long-lasting potentiation of auditory responses (including auditory-evoked potentials and auditory-evoked spike activities) in the ACx could be induced by high-frequency activation of ACC cell bodies or ACC-projecting terminals in the ACx. To our surprise, only the activation of ACC cell bodies caused long-term enhancement of auditory responses. Hence, we hypothesized that the ACC induces this effect through other brain areas. As previous studies show that the temporal cortex, specifically the rhinal cortex (RCx), possesses the ability to facilitate the formation of LTP and associative memory in the neocortex (X. Chen et al., 2013, 2019; Li et al., 2014; Doron et al., 2020; Z. Zhang et al., 2020; Lu et al., 2022), we further hypothesized that the ACC induces enhancement in the ACx by activating the RCx. To test this hypothesis, we conducted a series of physiological, optogenetic, and anatomic experiments. We also examined whether this effect is observed behaviorally.

## Materials and Methods

### Animals

All experimental procedures were approved by the Animal Subjects Ethics Sub-Committee of the City University of Hong Kong. Electrophysiological and anatomic experiments were conducted using adult male and female C57BL/6J mice. Behavioral experiments were conducted using only adult male C57BL/6J mice, considering that female mouse behavior could be influenced by the estrous cycle. Animals were recruited at ages from 8 to 12 weeks. Animals were housed under a 12 h dark/12 h light cycle (light on from 8:00-20:00 for animals in electrophysiological and anatomic experiments; light on from 20:00-8:00 for animals in behavioral experiments) at a stable temperature (23°C-25°C) with *ad libitum* access to food and water. Behavioral experiments were conducted during the dark cycle.

### Viruses

Adeno-associated viruses (AAVs) were purchased from Addgene (AAV9-Syn-ChrimsonR-tdTomato [#59171-AAV9], AAV8-Syn-DIO-mCherry [#50459-AAV8], AAV9-CaMKIIα-eGFP [#105541-AAV9], AAVretro-hSyn-Cre-WPRE-hGH [#105553-AAVrg]), BrainVTA (AAV9-hSyn-tdTomato-WPRE-hGHpA [#PT-1206-AAV9]) and Taitool Bioscience (AAV9-CaMKIIα-Chronos-GFP [#S0188-AAV9], AAV1-hSyn-Flpo-WPRE-pA [#S0271-AAV1], and AAV9-hEF1a-fDIO-eYFP-WPRE-pA [#S0253-AAV9]).

### *In vivo* electrophysiological experiments

#### Virus injection

For mice in electrophysiological experiments, AAV9-Syn-ChrimsonR-tdTomato (4.7E + 12 vg/ml) or AAV9-hSyn-tdTomato-WPRE-hGHpA (5.12E + 12 vg/ml) was injected into two locations of the aACC in the left hemisphere (AP 2.25 [site 1] and 2.55 [site 2] mm, ML 0.4 mm, DV −0.5 mm from dura, 200 nl per site), and AAV9-CaMKIIα-Chronos-GFP (5.6E + 12 vg/ml) or AAV9-CaMKIIα-eGFP (4.7E + 12 vg/ml) was injected into two locations of the lateral RCx (lRCx) in the left hemisphere (AP −4.2 mm, ML 4.1 mm, DV −3.8 [site 1] and −3.5 [site 2] mm from dura, 300 nl per site).

Before surgery, animals were administered intraperitoneal pentobarbital sodium (80 mg/kg, Dorminal 20%, Alfasan International). An animal was fixed in a stereotaxic device (RWD), and the scalp was incised after cleaning twice with 75% alcohol. The skull was washed with saline and then adjusted in position to level Bregma and Lambda sites as well as left and right sides. Craniotomies with diameters of ∼0.5-1 mm were created above the injection sites. AAV was loaded in a pipette with a fine tip mounted on the Nanoliter 2000/Micro4 system (World Precision Instruments). The injection pipette was slowly advanced to the target depth and paused for 5 min before the infusion. The infusion speed was 25 nl/min for aACC injection and 50 nl/min for lRCx injection. The pipette was slowly withdrawn 10 min after the infusion was complete. Four to 5 weeks were allowed for the expression of the virus.

#### Auditory stimuli and laser stimulation

White noise (Gaussian noise, frequency range 0-12.5 kHz with 10 ms raising and falling time) stimuli were digitally generated by Synapse controlled RZ6 processor (Tucker-Davis Technologies, sampling rate at 25 kHz) and delivered through a free-field magnetic speaker (MF-1, Tucker-Davis Technologies). The sound pressure level was calibrated with a sound level meter (RS PRO RS-8852 Sound Level Meter with Datalogger) with the A-weighing curve. The meter faced the speaker at a distance of 10 cm during measurement. During the experiment, the speaker was fixed at the same place facing the animal's right ear and 10 cm away. To choose a proper sound intensity that induced mild auditory responses, we first roughly determined the auditory response threshold in the mouse ACx by presenting noise stimuli at various sound intensities (40, 50, 60, 70, 80, and 90 dB, 10 trials each). The threshold was defined as the intensity at which ACx neurons started to show obvious auditory responses on examination of their raster plots. We selected an intensity 10 dB higher than the threshold to ensure that reliable auditory responses were evoked with sufficient room for modulation by manipulations. Laser stimulation was generated by a laser source (Inper #B1465635 Hangzhou) triggered by the Synapse-controlled RZ5D processor (Tucker-Davis Technologies). A fiber optic cannula (outer diameter 200 μm, 0.64 NA, Plexon) connected to an optical fiber patch cable was used to deliver the laser to the target area. A train of 40 laser pulses with 5 ms duration at 40 Hz was adopted as the high-frequency laser stimulation (HFLaser) protocol, whereas a train of 40 laser pulses with 5 ms duration at 1 Hz was adopted as the low-frequency laser stimulation (LFLaser) protocol. The laser power was 5 mW for neuronal cell body activation and 20 mW for neuronal projection activation.

#### Extracellular recording

For extracellular recordings in the ACx and lRCx, mice were first injected with urethane sodium (2 g/kg, i.p.), and anesthesia was maintained with periodic supplements throughout the surgery and neuronal recordings. A heating pad was used to keep the core body temperature of animals at 37°C. Mice were mounted in a stereotaxic device, and a midline incision was made in the scalp. The muscles above the ACx and lRCx were removed, after which a craniotomy was performed above the ACx. For the lRCx, the craniotomy was performed at the juncture of the temporal, occipital, and interparietal bones to expose the caudal rhinal vein and transverse sinus (G. W. Zhang et al., 2018). A 32-channel silicon probe (Cambridge NeuroTech #ASSY-37E) with two shanks (250 μm between shanks) attached to a λ-b fiber (100 mm core, 0.7 mm taper, 0 mm offset) or a four-shank 32-channel silicon probe (NeuroNexus #A4x8-5 mm, 100-200-177-A32, 200 μm between shanks) was inserted into ACx (tip depth at ∼800 μm) to record field EPSPs (fEPSPs) and multiunit activities (MUAs). Detected electrical signals were sequentially passed through the head stage (ZC32) and pre-amplifier (PZ5) to the acquisition processor (RZ5D) with a sampling rate of 25 kHz. The filter bandwidth was set as 300-5000 Hz for spike activity, and the threshold was set as 5 times of the SD above baseline to distinguish spikes. The filter bandwidth for fEPSPs was set as 3-300 Hz. Synapse software (Synapse suite, Tucker-Davis Technologies) was used to store the digitized data and control the RZ6 Multi I/O processor to present different stimuli to the animal. Electrode tracks were marked by DiI cell-labeling solution (Fisher Scientific #D282). During the entire recording, white noise (100 ms, 10 dB above the auditory response threshold, intertrial interval at 2 s) was presented continuously. The HFLaser or LFLaser protocol was applied after a baseline recording of 20 min, and auditory responses in terms of fEPSPs and MUAs were monitored for another hour.

For aACC neuronal responses to laser stimulation, to avoid any photoelectric artifacts, MUAs evoked by laser stimulation were recorded by glass pipette electrodes with an impedance of 1 m. Glass capillaries (TW 100-3, World Precision Instruments) were pulled using a micropipette puller (P-87, Sutter Instruments) with proper parameters. The impedance of the glass pipette electrode filled with 1 m NaCl was tested with a Multiclamp 700B microelectrode amplifier (Molecular Devices). A fiber optic cannula was held just on the surface of the aACC to present 40 pulses of laser stimulation at different frequencies (20, 40, 60, 80, 120, and 160 Hz) every 10 s.

#### Neural data analysis

For MUAs, a 5 ms time bin was adopted to calculate peristimulus time histograms (PSTHs). The response window was 0-0.1 s after the noise onset. Responses were averaged every 10 trials and then normalized as the percentage of change referencing each channel's mean response before laser stimulation. For fEPSPs, we recorded only one site (the deepest site) from each shank as a representative site considering the similar fEPSP origin for one cortical column. fEPSPs were averaged every 30 trials. The slope was measured by fitting a line to the initial linear phase of the fEPSP response. Slopes were normalized as the percentage of change referencing each site's mean slope before laser stimulation. All MUA and fEPSP signals were extracted and processed with MATLAB (The MathWorks) and then analyzed and visualized with R software (R Foundation).

#### Local drug infusion

A 10 µl syringe (Hamilton) coupled with a plastic tube and corresponding injector (RWD) was controlled using a syringe pump (KD Scientific) to infuse muscimol (600 nl, 1.5 mm, Sigma-Aldrich #M1523) into the left lateral RCx or lidocaine (300 nl, 2%, Tokyo Chemical Industry #L0156) into the left aACC. To label the diffusion range, we injected fluorescent muscimol (Fisher Scientific #M23400) into the lRCx and fluorescein (10 μm, J&K Scientific #916551) (Albani, 2004) into the aACC region. DiI dye solution (Fisher Scientific #D282) was used to confirm the electrode tracks after electrophysiological experiments.

### Anatomical experiments

#### Virus injection

For retrograde tracing, cholera toxin subunit B (CTB) with AlexaFluor-488 conjugate (CTB488, 5 mg/ml, Fisher Scientific #C34775) was injected into the left ACx (AP −1.8 [site 1], −2.3 [site 2], and −2.8 [site 3] mm, 0.5 mm under the edge differentiating the parietal skull and temporal skull, DV −0.5 mm from the dura, 200 nl for each site). CTB with AlexaFluor-647 conjugate (CTB647, 5 mg/ml, Fisher Scientific #C34778) was injected in the left lRCx (AP −4.2 mm, ML 4.1 mm, DV −3.8 [site 1] and −3.5 [site 2] mm, 350 nl for each site).

For collateral projection labeling, AAVretro-hSyn-Cre-WPRE-hGH (1.83 + 13 vg/ml) was injected into the ACx (AP −1.8 [site 1], −2.3 [site 2], and −2.8 [site 3] mm, 0.5 mm under the edge differentiating the parietal skull and temporal skull, DV −0.5 mm from the dura, 200 nl for each site). After 1 week, AAV8-Syn-DIO-mCherry (5.3 + 12 vg/ml) was injected into the aACC (AP 2.25 [site 1] and 2.55 [site 2] mm, ML 0.4 mm, DV −0.5 mm from the dura, 200 nl for each site).

For anterograde tracing, the left aACC (the same coordinates mentioned above) was injected with AAV1-hSyn-Flpo-WPRE-pA (1.92E + 13 vg/ml, 200 nl for each site). After 1 week, the left lRCx (the same coordinates mentioned above) was injected with AAV9-hEF1a-fDIO-eYFP-WPRE-pA (1.76E + 13 vg/ml, 350 nl for each site).

#### Histology

Mice were deeply anesthetized with pentobarbital sodium (50 mg/kg, i.p.) and sequentially perfused with 30 ml of 1× PBS followed by 30 ml of 4% (w/v) PFA. The brains were subsequently removed and postfixed in 4% PFA at 4°C overnight. After cryoprotection of the brains with 30% (w/v) sucrose, coronal sections (70 μm) were cut on a cryostat (Fisher Scientific #HM525NX). The brain slices were mounted on a glass slide with 70% glycerol in PBS, and fluorescent images were taken by a Nikon Eclipse Ni-E upright fluorescence microscope (4×, Nikon) and confocal microscope (20×, Nikon). For imaging signal analysis, including quantification of neuron number and projection intensity, we used Fiji software (https://imagej.net/Fiji) (Schindelin et al., 2012). To calculate the number (and percentage) of CTB retrograde somas in the aACC region, we selected half of the coronal slices indiscriminately from each subject (four images for each mouse). In the aACC region, we defined layers 1-3 as superficial layers and layers 5-6 as deep layers according to the Allen Mouse Brain Atlas (https://atlas.brain-map.org/). The Cell Counter plugin in Fiji was used to count cell numbers. To measure the projection intensity of ACC neural fibers, the FeatureJ plugin was used in Fiji. Fiber-like signals were extracted using a Hessian filter, and the fewest eigenvalues were selected when converting the raw images to Eigen images. The intensity of neural projections was assessed as the pixel density after converting Eigen images to binary images in Fiji (Grider et al., 2006).

### Behavioral experiments

#### Virus injection and implantation of optical fibers

AAV9-Syn-ChrimsonR-tdTomato or AAV9-hSyn-tdTomato-WPRE-hGHpA was injected into the left aACC, and 4-5 weeks were allowed for virus expression. On the optical fiber implantation day, dexamethasone (2 mg/kg, i.p.) and carprofen (5 mg/kg, i.p.) were administered at least 1 h before anesthetization induced by pentobarbital sodium (80 mg/kg). A mouse was then fixed in a stereotaxic device (RWD), and the scalp was incised. After cleaning and leveling the skull, small holes were drilled bilaterally to reach the lRCx. The dura was removed, and optical fibers (outer diameter 200 μm for optic cannula, 0.37 NA, Plexon) were slowly inserted. Kwik-cast gel (World Precision Instruments) was applied around the cannula to seal the drilled skull. A thin layer of adhesive cement (C&B Metabond, Parkell) was then applied to the skull. Fibers were fixed to the skull with dental cement (mega PRESS NV + JET X, Megadental). Finally, a long screw was attached to the skull (45° to the vertical axis) with dental cement for later head-fixed experiments.

#### Behavioral task

We used a sound-evoked flight behavioral paradigm (Xiong et al., 2015; Sun et al., 2022). Animals were head-fixed on a flat plate that rotated smoothly around its center, with speed detected by a rotary encoder and recorded in real time. After animals were habituated to the setup for 2 d, two blocks of behavioral sessions began. In the first baseline block, after habituation to the plate for 10 min, head-fixed mice were presented with 40 white noise stimulations (30, 50, 70, and 90 dB, 5 s duration, repeated 10 times at each intensity) by a speaker placed 10 cm above the animal's head with a random 50-70 s intertrial interval. The white noise stimuli were generated and calibrated as described in Auditory stimuli and laser stimulation. The HFLaser protocol (647 nm, 40 pulses at 40 Hz, 5 ms duration, starting 0.5 s before the onset of the sound, 20 mW) was paired with the white noise (30, 50, 70, and 90 dB, 5 s duration, once at each intensity) 4 times with a random 50-70 s intertrial interval. The mice were then returned to their home cage. One hour later, in the second testing block, mice were head-fixed on the plate and presented with the same 40 white noises. We assessed running performance over 3 or 4 sessions for each mouse. Running speed was recorded and analyzed by MATLAB.

### Statistical analysis

Group data are shown as mean ± SEM unless otherwise stated. Statistical analyses, including paired sample *t* tests, two-way repeated ANOVA, and two-way mixed ANOVA, were conducted in SPSS 26 (IBM). Statistical significance was defined as *p* < 0.05 by default (for detailed statistics, see Table 1).

**Table 1.** Statistics for Figures 1–7

Figure	Statistic test	Exact *p* value, *F* value with degree of freedom for ANOVAs, *t* value with degree of freedom (df) for *t* test, *k* (multiple comparison number), *n* (sample size)
1 *D*	One-way repeated-measures ANOVA	Correction for sphericity violation: Greenhouse–Geisser
		Manipulation *F*_(2.372, 30.837)_ =14.726, *p* < 0.001, *n* = 14 sites in *N* = 3 animals
		*Post hoc* test: paired *t* test
		Adjustment for multiple comparisons: Bonferroni, *k* = 15
		20 Hz in aACC (ChrimsonR) vs 40 Hz in aACC (ChrimsonR), 1.779 ± 0.138 vs 1.655 ± 0.137, *t* = 2.913, df = 13, *p* = 0.012, after adjustment, *p* = 0.18
		20 Hz in aACC (ChrimsonR) vs 60 Hz in aACC (ChrimsonR), 1.779 ± 0.138 vs 1.508 ± 0.138, *t* = 3.700, df = 13, *p* = 0.003, after adjustment, *p* = 0.045
		20 Hz in aACC (ChrimsonR) vs 80 Hz in aACC (ChrimsonR), 1.779 ± 0.138 vs 1.279 ± 0.135, *t* = 5.198, df = 13, *p* < 0.001, after adjustment, *p* < 0.001
		20 Hz in aACC (ChrimsonR) vs 120 Hz in aACC (ChrimsonR), 1.779 ± 0.138 vs 1.112 ± 0.156, *t* = 4.705, df = 13, *p* < 0.001, after adjustment, *p* < 0.001
		20 Hz in aACC (ChrimsonR) vs 160 Hz in aACC (ChrimsonR), 1.779 ± 0.138 vs 1.099 ± 0.118, *t* = 4.776, df = 13, *p* < 0.001, after adjustment, *p* < 0.001
2 *K*	Two-way mixed ANOVA	Correction for sphericity violation: Greenhouse–Geisser
		Manipulation × Groups *F*_(3, 1191)_ = 121.113, *p* < 0.001
		Manipulation *F*_(1, 1191)_ = 97.932, *p* < 0.001
		Group *F*_(3, 1191)_ = 88.936, *p* < 0.001
		*Post hoc* test: paired *t* test
		Adjustment for multiple comparisons: Bonferroni, *k* = 1
		LFLaser in aACC (ChrimsonR): before vs after, 99.9 ± 0.7% vs 100.3 ± 1.6%, *t* = −0.354, df = 331, *p* = 0.723, *n* = 332 sites in *N* = 11 animals
		HFLaser in aACC (ChrimsonR): before vs after, 101.3 ± 0.8% vs 163 ± 4.9%, *t* = −12.688, df = 305, *p* < 0.001, *n* = 306 sites in *N* = 11 animals
		HFLaser in aACC (tdTomato): before vs after, 99.9 ± 0.6% vs 102.7 ± 1.8%, *t* = −1.545, df = 215, *p* = 0.124, *n* = 216 sites in *N* = 5 animals
		HFLaser in ACx (ChrimsonR) activating aACC-to-ACx projections: before vs after, 105.3 ± 0.9% vs 99.1 ± 2.3%, *t* = 3.128, df = 340, *p* = 0.002, *n* = 341 sites in *N* = 11 animals
		*Post hoc* test: two-sample *t* test
		Adjustment for multiple comparisons: Bonferroni, *k* = 6
		HFLaser in aACC (ChrimsonR) + after vs LFLaser in aACC (ChrimsonR) + after, 163.0 ± 4.9% vs 100.3 ± 1.6%, *t* = 12.601, df = 636, *p* < 0.001, after adjustment, *p* < 0.001
		HFLaser in aACC (ChrimsonR) + after vs HFLaser in aACC (tdTomato) + after, 163.0 ± 4.9% vs 102.7 ± 1.8%, *t* = 10.0, df = 520, *p* < 0.001, after adjustment, *p* < 0.001
		HFLaser in aACC (ChrimsonR) + after vs HFLaser in ACx (ChrimsonR) activating aACC-to-ACx projections + after, 163.0 ± 4.9% vs 99.1 ± 2.3%, df = 645, *p* < 0.001, after adjustment, *p* < 0.001
2 *L*	Two-way mixed ANOVA	Correction for sphericity violation: Greenhouse–Geisser
		Manipulation × Groups *F*_(3, 104)_ = 29.071, *p* < 0.001
		Manipulation *F*_(1104)_ = 15.425, *p* < 0.001
		Group *F*_(3, 104)_ = 40.353, *p* < 0.001
		*Post hoc* test: paired *t* test
		Adjustment for multiple comparisons: Bonferroni, *k* = 1
		LFLaser in aACC (ChrimsonR): before vs after, 99.5 ± 0.8% vs 98.7 ± 3.0%, *t* = 0.270, df = 27, *p* = 0.789, *n* = 28 sites in *N* = 11 animals
		HFLaser in aACC (ChrimsonR): before vs after, 101.3 ± 1.0% vs 139.1 ± 4.8%, *t* = −7.054, df = 27, *p* < 0.001, *n* = 28 sites in *N* = 11 animals
		HFLaser in aACC (tdTomato): before vs after, 99.2 ± 0.5% vs 94.1 ± 2.8%, *t* = 1.806, df = 23, *p* = 0.084, *n* = 24 sites in *N* = 5 animals
		HFLaser in ACx (ChrimsonR) activating aACC-to-ACx projections: before vs after, 100.6 ± 1.2% vs 98.6 ± 2.9%, *t* = 0.648, df = 27, *p* = 0.522, *n* = 28 sites in *N* = 11 animals
		*Post hoc* test: two-sample *t* test
		Adjustment for multiple comparisons: Bonferroni, *k* = 6
		HFLaser in aACC (ChrimsonR) + after vs LFLaser in aACC (ChrimsonR) + after, 139.1 ± 4.8% vs 98.7 ± 3.0%, *t* = 7.146, df = 54, *p* < 0.001, after adjustment, *p* < 0.001
		HFLaser in aACC (ChrimsonR)+ after vs HFLaser in aACC (tdTomato) + after, 139.1 ± 4.8% vs 94.1 ± 2.8%, *t* = 7.733, df = 50, *p* < 0.001, after adjustment, *p* < 0.001
		HFLaser in aACC (ChrimsonR) + after vs HFLaser in ACx (ChrimsonR) activating aACC-to-ACx projections + after, 139.1 ± 4.8% vs 98.6 ± 2.9%, *t* = 7.220, df = 53, *p* < 0.001, after adjustment, *p* < 0.001
3 *H*	Two-way mixed ANOVA	Correction for sphericity violation: Greenhouse–Geisser
		Manipulation × Groups *F*_(1, 496)_ = 51.229, *p* < 0.001
		Manipulation *F*_(1496)_ = 64.305, *p* < 0.001
		Group *F*_(1, 496)_ = 51.614, *p* < 0.001
		*Post hoc* test: paired *t* test
		Adjustment for multiple comparisons: Bonferroni, *k* = 1
		HFLaser in ACx (Chronos) activating lRCx-to-ACx projections: before vs after, 100.6 ± 0.5% vs 135.2 ± 3.6%, *t* = −9.698, df = 272, *p* < 0.001, *n* = 273 sites in *N* = 6 animals
		HFLaser in ACx (eGFP) activating lRCx-to-ACx projections: before vs after, 99.1 ± 0.8% vs 101.1 ± 2.7%, *t* = −0.771, df = 224, *p* = 0.441, *n* = 225 sites in *N* = 6 animals
		*Post hoc* test: two-sample *t* test
		Adjustment for multiple comparisons: Bonferroni, *k* = 1
		HFLaser in ACx (Chronos) activating lRCx-to-ACx projections + after vs HFLaser in ACx (eGFP) activating lRCx-to-ACx projections + after, 135.2 ± 3.6% vs 99.6 ± 2.7%, *t* = 7.625, df = 491, *p* < 0.001
3 *I*	Two-way mixed ANOVA	Correction for sphericity violation: Greenhouse–Geisser
		Manipulation × Groups *F*_(1, 46)_ = 25.035, *p* < 0.001
		Manipulation *F*_(1,46)_ = 10.957, *p* < 0.001
		Group *F*_(1, 46)_ = 14.102, *p* < 0.001
		*Post hoc* test: paired *t* test
		Adjustment for multiple comparisons: Bonferroni, *k* = 1
		HFLaser in ACx (Chronos) activating lRCx-to-ACx projections: before vs after, 100.1 ± 1.5% vs 141.9 ± 10.2%, *t* = −4.385, df = 23, *p* < 0.001, *n* = 24 sites in *N* = 6 animals
		HFLaser in ACx (eGFP) activating lRCx-to-ACx projections: before vs after, 103.2 ± 1.6% vs 94.7 ± 3.0%, *t* = 2.659, df = 23, *p* = 0.014, *n* = 24 sites in *N* = 6 animals
		*Post hoc* test: two-sample *t* test
		Adjustment for multiple comparisons: Bonferroni, *k* = 1
		HFLaser in ACx (Chronos) activating lRCx-to-ACx projections + after vs HFLaser in ACx (eGFP) activating lRCx-to-ACx projections + after, 141.9 ± 10.2% vs 94.7 ± 3.0%, *t* = 4.411, df = 46, *p* < 0.001
5 *E*	Paired *t* test	HFLaser in aACC (ChrimsonR) and Mus. in lRCx: before vs after, 99.8 ± 0.5% vs 96.0 ± 1.6%
		*t* = 2.577, df = 267, *p* = 0.01, *n* = 268 sites in *N* = 11 animals
5 *G*	Paired *t* test	HFLaser in aACC (ChrimsonR) and Mus. in lRCx: before vs after, 99.8 ± 1.11% vs 101.6 ± 3.5%
		*t* = −0.564, df = 23, *p* = 0.578, *n* = 24 sites in *N* = 11 animals
6 *H*	Two-way mixed ANOVA	Correction for sphericity violation: Greenhouse–Geisser
		Manipulation × Groups *F*_(1, 390)_ = 57.335, *p* < 0.001
		Manipulation *F*_(1, 390)_ = 52.225, *p* < 0.001
		Group *F*_(1, 390)_ = 60.493, *p* < 0.001
		*Post hoc* test: paired *t* test
		Adjustment for multiple comparisons: Bonferroni, *k* = 1
		HFLaser in lRCx (ChrimsonR) activating aACC-to-lRCx projections: before vs after, 108.3 ± 1.5% vs 155.7 ± 6.1%, *t* = −8.097, df = 202, *p* < 0.001, *n* = 203 sites in *N* = 7 animals
		HFLaser in lRCx (tdTomato) activating aACC-to-lRCx projections: before vs after, 102.7 ± 1.4% vs 101.6 ± 2.5%, *t* = 0.519, df = 188, *p* = 0.604, *n* = 189 sites in *N* = 7 animals
		*Post hoc* test: two-sample *t* test
		Adjustment for multiple comparisons: Bonferroni, *k* = 1
		HFLaser in lRCx (ChrimsonR) activating aACC-to-lRCx projections + after vs HFLaser in lRCx (tdTomato) activating aACC-to-lRCx projections + after: 155.7 ± 6.1% vs 101.6 ± 2.5%, *t* = 7.992, df = 390, *p* < 0.001
6 *I*	Two-way mixed ANOVA	Correction for sphericity violation: Greenhouse–Geisser
		Manipulation × Groups *F*_(1, 46)_ = 31.642, *p* < 0.001
		Manipulation *F*_(1,46)_ = 30.970, *p* < 0.001
		Group *F*_(1, 46)_ = 37.992, *p* < 0.001
		*Post hoc* test: paired *t* test
		Adjustment for multiple comparisons: Bonferroni, *k* = 1
		HFLaser in lRCx (ChrimsonR) activating aACC-to-lRCx projections: before vs after, 101.8 ± 1.2% vs 138.9 ± 5.4%, *t* = −6.572, df = 23, *p* < 0.001, *n* = 24 sites in *N* = 7 animals
		HFLaser in lRCx (tdTomato) activating aACC-to-lRCx projections: before vs after, 99.6 ± 1.2% vs 99.4 ± 3.6%, *t* = 0.057, df = 23, *p* = 0.955, *n* = 24 sites in *N* = 7 animals
		*Post hoc* test: two-sample *t* test
		Adjustment for multiple comparisons: Bonferroni, *k* = 1
		HFLaser in lRCx (ChrimsonR) activating aACC-to-lRCx projections + after vs HFLaser in lRCx (tdTomato) activating aACC-to-lRCx projections + after, 138.9 ± 5.4% vs 99.4 ± 3.6%, *t* = 6.901, df = 46, *p* < 0.001
7 *C*	Paired *t* test	HFLaser in lRCx (ChrimsonR) activating aACC-to-lRCx projections:
		before vs after, 9.97 ± 0.89 vs 12.99 ± 0.83
		*t* = −5.650, df = 22, *p* < 0.001
		*n* = 23 sessions in *N* = 7 animals
7 *D*	Two-way repeated ANOVA	Correction for sphericity violation: Greenhouse–Geisser
		Manipulation × sound intensity *F*_(2.641, 58.092)_ = 2.244, *p* = 0.1
		Manipulation *F*_(1,22)_ = 28.193, *p* < 0.001
		Sound intensity *F*_(2.397, 52.724)_ = 88.380, *p* < 0.001
		Manipulation × sound intensity:
		*Post hoc* test: paired *t* test
		Adjustment for multiple comparisons: Bonferroni, *k* = 1
		HFLaser in lRCx (ChrimsonR) activating aACC-to-lRCx projections:
		Sound intensity at 30 dB: before vs after, 0.01 ± 0.26 vs 1.13 ± 0.46, *t* = −2.103, df = 22, *p* = 0.047
		Sound intensity at 50 dB: before vs after, 3.03 ± 0.74 vs 4.80 ± 0.97, *t* = −2.497, df = 22, *p* = 0.021
		Sound intensity at 70 dB: before vs after, 7.58 ± 0.97 vs 8.99 ± 0.92, *t* = −2.383, df = 22, *p* = 0.026
		Sound intensity at 90 dB: before vs after, 9.97 ± 0.89 vs 12.99 ± 0.83, *t* = −5.650, df = 22, *p* < 0.001
		Manipulation:
		*Post hoc* test: paired *t* test
		Adjustment for multiple comparisons: Bonferroni, *k* = 1
		before vs after, 5.15 ± 0.55 vs 6.98 ± 0.62, *t* = −6.044, df = 91, *p* < 0.001
		*n* = 23 sessions in *N* = 7 animals
7 *E*	Paired *t* test	HFLaser in lRCx (tdTomato) activating aACC-to-lRCx projections:
		before vs after, 12.90 ± 1.23 vs 11.33 ± 1.24
		*t* = 2.081, df = 21, *p* = 0.05
		*n* = 22 sessions in *N* = 7 animals
7 *F*	Two-way repeated ANOVA	Correction for sphericity violation: Greenhouse–Geisser
		Manipulation × sound intensity *F*_(2.843, 59.693)_ = 0.513, *p* = 0.597
		Manipulation *F*_(1,21)_ = 3.802, *p* = 0.065
		Sound intensity *F*_(1.419, 29.795)_ = 73.764, *p* < 0.001
		Manipulation × sound intensity:
		*Post hoc* test: paired *t* test
		Adjustment for multiple comparisons: Bonferroni, *k* = 1
		HFLaser in lRCx (tdTomato) activating aACC-to-lRCx projections:
		Sound intensity at 30 dB: before vs after, −0.19 ± 0.31 vs −0.41 ± 0.45, *t* = 0.375, df = 21, *p* = 0.712
		Sound intensity at 50 dB: before vs after, 2.65 ± 0.66 vs 1.54 ± 0.51, *t* = 1.396, df = 21, *p* = 0.177
		Sound intensity at 70 dB: before vs after, 7.16 ± 1.30 vs 6.43 ± 1.05, *t* = 0.763, df = 21, *p* = 0.454
		Sound intensity at 90 dB: before vs after, 12.90 ± 1.23 vs 11.33 ± 1.24, *t* = 2.081, df = 21, *p* = 0.05
		Manipulation:
		*Post hoc* test: paired *t* test
		Adjustment for multiple comparisons: Bonferroni, *k* = 1
		before vs after, 5.63 ± 0.71 vs 4.72 ± 0.65, *t* = 2.334, df = 87, *p* = 0.022
		*n* = 22 sessions in *N* = 7 animals

## Results

### High-frequency activation of aACC cell bodies, but not aACC-to-ACx-projecting terminals, induces long-term enhancement of auditory responses in the ACx

**Figure 1.**
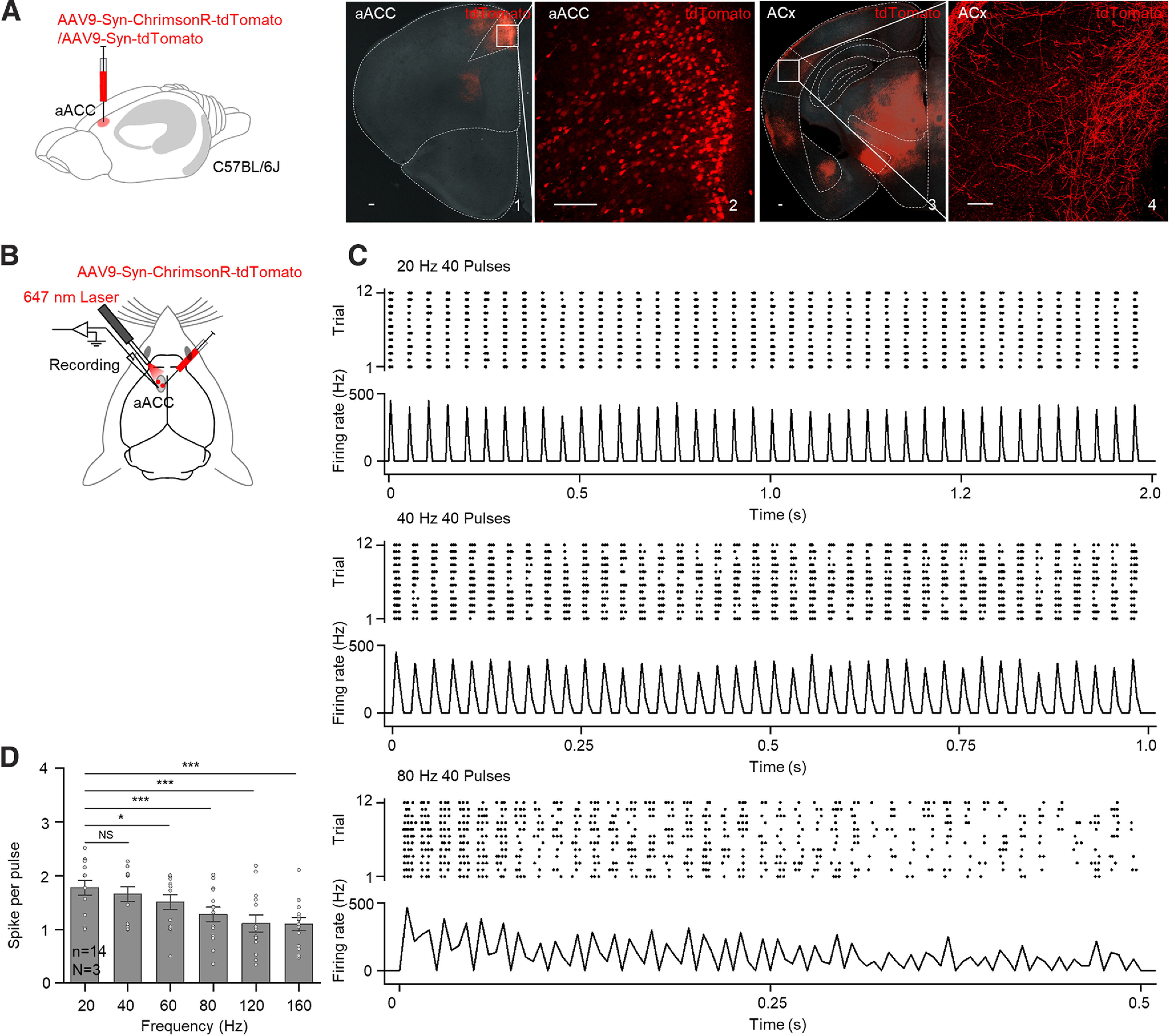
aACC neuron activities followed high-frequency laser stimulation up to 40 Hz. ***A***, Left, Schematic drawing showing AAV9-Syn-ChrimsonR-tdTomato or AAV9-Syn-tdTomato injected into the aACC. Right, Representative images showing tdTomato expression in the injection site (1 and 2; scale bar, 100 µm) and ACx (3 and 4; scale bar, 100 µm). ***B***, Schematic diagram showing the experimental setup. ***C***, Raster and PSTH plots showing aACC neuron responses to 20, 40, and 80 Hz laser stimulation. ***D***, aACC neuron's average spike number during each laser pulse at different frequencies (20, 40, 60, 80, 120, and 160 Hz). *n* represents the total number of recording sessions. *N* represents the number of animals recruited in the experiment. Error bars represent SEM. *p < 0.05; **p < 0.01; ***p < 0.001; NS, no significant changes; one-way repeated-measures ANOVA (for detailed statistics, see Table 1).

**Figure 2.**
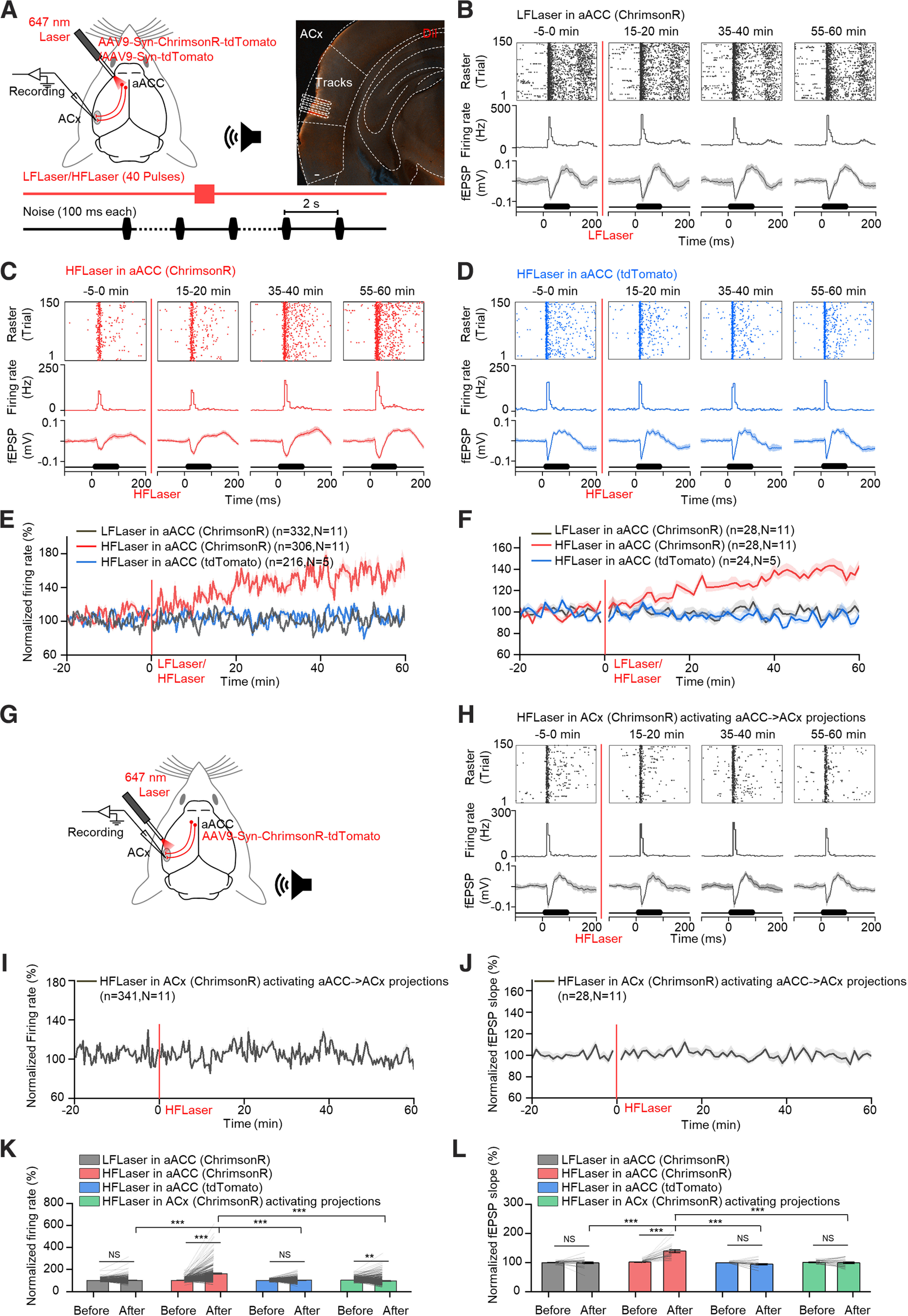
High-frequency activation of the aACC, but not aACC-to-ACx-projecting terminals, induced long-term enhancement of auditory responses in the ACx. ***A***, Left, Schematic diagram represents the experimental setup and protocol. Right, Representative image showing the tracks generated by multiple penetrations of the array inserted into the ACx. Scale bar, 100 µm. ***B***, Auditory responses from a representative recording site (top: raster plots; middle: PSTHs; bottom: fEPSPs) at different time points (−5 to 0, 15-20, 35-40, and 55-60 min) relative to the LFLaser protocol applied at the aACC in mice injected with AAV9-Syn-ChrimsonR-tdTomato in the aACC (Condition 1). ***C***, Same as in ***B***, except for the HFLaser protocol was applied at the aACC in mice injected with AAV9-Syn-ChrimsonR-tdTomato in the aACC (Condition 2). ***D***, Same as in ***B***, except for the HFLaser protocol was applied at the aACC in mice injected with AAV9-Syn-tdTomato (control virus) in the aACC (Condition 3). ***E***, Average normalized firing rate tracings in Condition 1 (black curve), Condition 2 (red curve), and Condition 3 (blue curve). ***F***, Average normalized fEPSP slope tracings for conditions shown in ***E***. ***G***, Schematic diagram showing the experimental setup. ***H***, Same as in ***B***, except for the HFLaser protocol was applied at the ACx in mice injected with AAV9-Syn-ChrimsonR-tdTomato in the ACC (Condition 4). ***I***, Average normalized firing rate tracing in Condition 4. ***J***, Average normalized fEPSP slope tracing in Condition 4. ***K***, Average and individual normalized firing rates before (−10 to 0 min) and after (50-60 min) the LFLaser or HFLaser protocol in Conditions 1-4. ***L***, Average and individual normalized fEPSP slopes before (−10 to 0 min) and after (50-60 min) the LFLaser or HFLaser protocol in Conditions 1-4. *n* represents the total number of recording sessions. *N* represents the number of animals recruited in the experiment. Shadows and error bars represent SEM. *p < 0.05; **p < 0.01; ***p < 0.001; NS, no significant changes; two-way mixed ANOVA (for detailed statistics, see Table 1).

Our recent study shows that a single pulse activation of aACC terminals in the ACx immediately potentiates auditory responses in the ACx (Sun et al., 2022). Here, to investigate a possible long-term effect, an optogenetic virus (AAV9-Syn-ChrimsonR-tdTomato) expressing a red light-sensitive cation channel (ChrimsonR) that can be activated by a 647 nm laser was injected into the aACC of WT mice. Five weeks after virus injection, strong expression of tdTomato was detected in both the injection site (aACC) and the ACx (Fig. 1*A*). To confirm the effectiveness of the virus and responsiveness of aACC neurons, a glass pipette electrode along with an optical fiber was inserted into the virus injection region of anesthetized mice (Fig. 1*B*). Forty laser pulses at different frequencies (20, 40, 60, 80, 120, and 160 Hz) were applied. aACC neuron responses faithfully followed up to 40 Hz (Fig. 1*C*,*D*). Therefore, the HFLaser protocol (forty 647 nm laser pulses at 40 Hz) was used to strongly activate the aACC, and the LFLaser protocol (forty 647 nm laser pulses at 1 Hz) was used as a control. An electrode array was inserted into the ACx to record fEPSPs and MUAs, and an optical fiber was placed at the aACC to shine the laser stimuli (Fig. 2*A*). A series of white noises (100 ms, intertrial interval at 2 s) was presented. To identify the auditory response threshold in the ACx, neuronal responses to 100 ms noise stimuli at different sound intensities (40, 50, 60, 70, 80, and 90 dB, 10 trials each) were examined. We selected an intensity 10 dB higher than the threshold to ensure that reliable auditory responses were evoked, and there was sufficient room for modulation following manipulations. After a baseline recording of 20 min, the HFLaser or LFLaser protocol was applied, and auditory responses were monitored for another hour. The LFLaser protocol did not induce a significant change in auditory responses in terms of fEPSPs or MUAs (Fig. 2*B* for example recording site, Fig. 2*E*, black curve for temporal variation in averaged normalized firing rates of MUAs during noise presentation, Fig. 2*F*, black curve for temporal variation of averaged normalized fEPSP slopes, and Fig. 2*K*, *L*, gray bars for comparisons between −10-0 and 50-60 min after the LFLaser protocol for normalized firing rates [99.9 ± 0.7% vs 100.3 ± 1.6%] and normalized fEPSP slopes [99.5 ± 0.8% vs 98.7 ± 3.0%]). By contrast, after the HFLaser protocol, both the slopes of fEPSPs and firing rates of MUAs gradually increased (Fig. 2*C* for example recording site, Fig. 2*E*,*F*, red curves, and Fig. 2*K*,*L*, red bars, 101.3 ± 0.8% vs 163 ± 4.9% for normalized firing rates of MUAs and 101.3 ± 1.0% vs 139.1 ± 4.8% for normalized fEPSP slopes), indicating that only high-frequency activation of aACC neuronal cell bodies promoted long-term enhancement of auditory responses in the ACx. The slopes of fEPSPs started to increase earlier than the firing rates of MUAs, possibly because of the nonlinear relationship between the input and output of neurons in the ACx (London et al., 2002; Mittmann and Häusser, 2007). In a control experiment in which a control virus (AAV9-Syn-tdTomato), which lacks the ChrimsonR light-activated cation channel, was injected into the same aACC region, the HFLaser protocol did not have any significant effect (Fig. 2*D* for example recording site, Fig. 2*E*,*F*, blue curve, and Fig. 2*K*,*L*, blue bars, 99.9 ± 0.6% vs 102.7 ± 1.8% for normalized firing rates of MUAs and 99.2 ± 0.5% vs 94.1 ± 2.8% for normalized fEPSP slopes).

To rule out the effect of other areas triggered by the stimulation of aACC neuronal cell bodies, in the next experiment, the optical fiber was shifted to the ACx to activate aACC-to-ACx-projecting terminals (Fig. 2*G*). To our surprise, the HFLaser protocol did not induce significant changes in either fEPSPs or MUAs in response to noises (Fig. 2*H* for example recording site, Fig. 2*I*,*J*, and Fig. 2*K*,*L* green bars, 105.3 ± 0.9% vs 99.1 ± 2.3% for normalized firing rates of MUAs and 100.6 ± 1.2% vs 98.6 ± 2.9% for normalized fEPSP slopes).

Therefore, once strongly activated, the aACC possessed the ability to potentiate auditory responses in the ACx in the long-term. As this effect was not mediated by direct projections from the aACC to the ACx, other areas were potentially involved.

### High-frequency activation of lateral RCx-projecting terminals in the ACx induces long-term enhancement of auditory responses in the ACx

**Figure 3.**
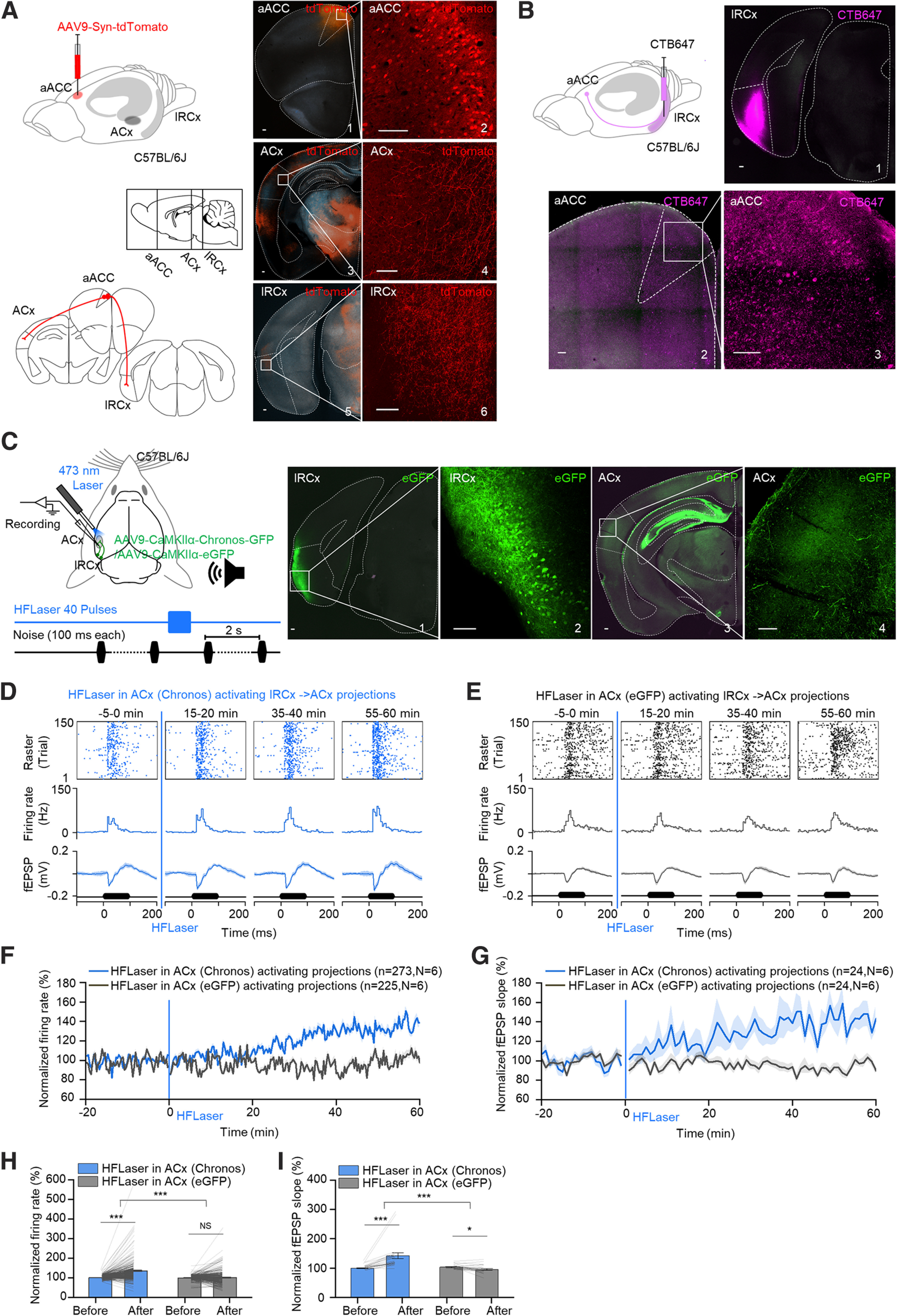
High-frequency activation of lRCx-to-ACx-projecting terminals in the ACx induced long-term enhancement of auditory responses in the ACx. ***A***, Left, Schematic drawing represents AAV9-Syn-tdTomato injected into the aACC and the relative position of the aACC, ACx, and lRCx. Right, Representative image showing tdTomato expression in the aACC (1 and 2), ACx (3 and 4), and lRCx (5 and 6). Scale bars, 100 µm. ***B***, Left, Schematic drawing showing CTB647 injected into the lRCx. Right, Representative images showing CTB signals in the lRCx (1) and aACC (2 and 3). Scale bars, 100 µm. ***C***, Left, Schematic drawing showing AAV9-CaMKIIα-Chronos-GFP or AAV9-CaMKIIα-eGFP injected into the lRCx. Right, Representative images showing eGFP expression in the injection site (1 and 2; scale bar, 100 µm) and ACx (3 and 4; scale bar, 100 µm). ***D***, Auditory responses from a representative recording site (top: raster plots; middle: PSTHs; bottom: fEPSPs) at different time points (−5 to 0, 15-20, 35-40, and 55-60 min) relative to the HFLaser protocol applied at the ACx in mice injected with AAV9-CaMKIIα-Chronos-GFP in the lRCx (Condition 1). ***E***, Same as in ***D***, except for the HFLaser protocol was applied at the ACx in mice injected with AAV9-CaMKIIα-eGFP (control virus) in the lRCx (Condition 2). ***F***, Average normalized firing rate tracings in Condition 1 (blue curve) and Condition 2 (black curve). ***G***, Average and individual normalized firing rates before (−10 to 0 min) and after (50-60 min) the HFLaser protocol in Conditions 1 (blue bar) and 2 (gray bar). ***H***, Average normalized fEPSP slope tracings for conditions shown in ***F***. ***I***, Average and individual normalized fEPSP slopes before (−10 to 0 min) and after (50-60 min) the HFLaser protocol for conditions shown in ***G***. *n* represents the total number of recording sessions. *N* represents the number of animals recruited in the experiment. Shadows and error bars represent SEM. *p < 0.05; **p < 0.01; ***p < 0.001; NS, no significant changes; two-way mixed ANOVA (for detailed statistics, see Table 1).

In our previous studies, we demonstrated that the RCx (entorhinal part) promotes the formation of LTP and auditory associative memory in the ACx through its cholecystokinin-positive projections (Li et al., 2014; X. Chen et al., 2019; Z. Zhang et al., 2020). Thus, we first examined whether the aACC projects to the RCx by injecting a virus (AAV9-Syn-tdTomato) into the aACC region (Fig. 3*A*). High levels of tdTomato signal were observed in both the ACx and lRCx regions, implying that the aACC induces long-term enhancement of auditory responses in the ACx by activating the lRCx. Retrograde labeling by CTB conjugated with a fluorescent tag Alexa-647 injected into the RCx further confirmed the existence of aACC-to-RCx projections (Fig. 3*B*). As we previously focused on the entorhinal part of the RCx, we next investigated whether the lRCx possesses a similar ability. A virus (AAV9-CaMKIIα-Chronos-GFP) expressing a blue light-sensitive cation channel (Chronos) that can be activated by a 473 nm laser was injected into the lRCx of WT mice to infect excitatory neurons through the CaMKIIα promoter (Fig. 3*C*). Strong expression of GFP was found in the ACx 4 weeks later (Fig. 3*C*). An electrode array together with an optical fiber was inserted in the ACx to record fEPSPs and MUAs, a train of forty 473 nm laser pulses at 40 Hz was applied in the ACx to activate the lRCx to ACx-projecting terminals. After the HFLaser protocol, similar to ACC neuronal cell body activation, a gradual increase in auditory responses in the ACx was observed (Fig. 3*D* for example recording site, Fig. 3*F*,*G*, blue curves, and Fig. 3*H*,*I*, blue bars, 100.6 ± 0.5% vs 135.2 ± 3.6% for normalized firing rates of MUAs and 100.1 ± 1.5% vs 141.9 ± 10.2% for normalized fEPSP slopes). Again, an increase in fEPSP slopes led to an increase in the firing rates of MUAs, implying the same underlying mechanism as ACC neuronal cell body activation. In a control experiment in which the virus without the Chronos light-activated cation channel (AAV9-CaMKIIα-eGFP) was injected, no significant change was observed after the HFLaser protocol (Fig. 3*E* for example recording site, Fig. 3*F*,*G*, black curves, and Fig. 3*H*,*I*, gray bars, 99.1 ± 0.8% vs 101.1 ± 2.7% for normalized firing rates of MUAs and 103.2 ± 1.6% vs 94.7 ± 3.0% for normalized fEPSP slopes). These results show that the lRCx is a potential site that endows the aACC with the ability to promote the long-term enhancement of auditory responses in the ACx.

### Two groups of aACC neurons separately project to the ACx and lRCx

**Figure 4.**
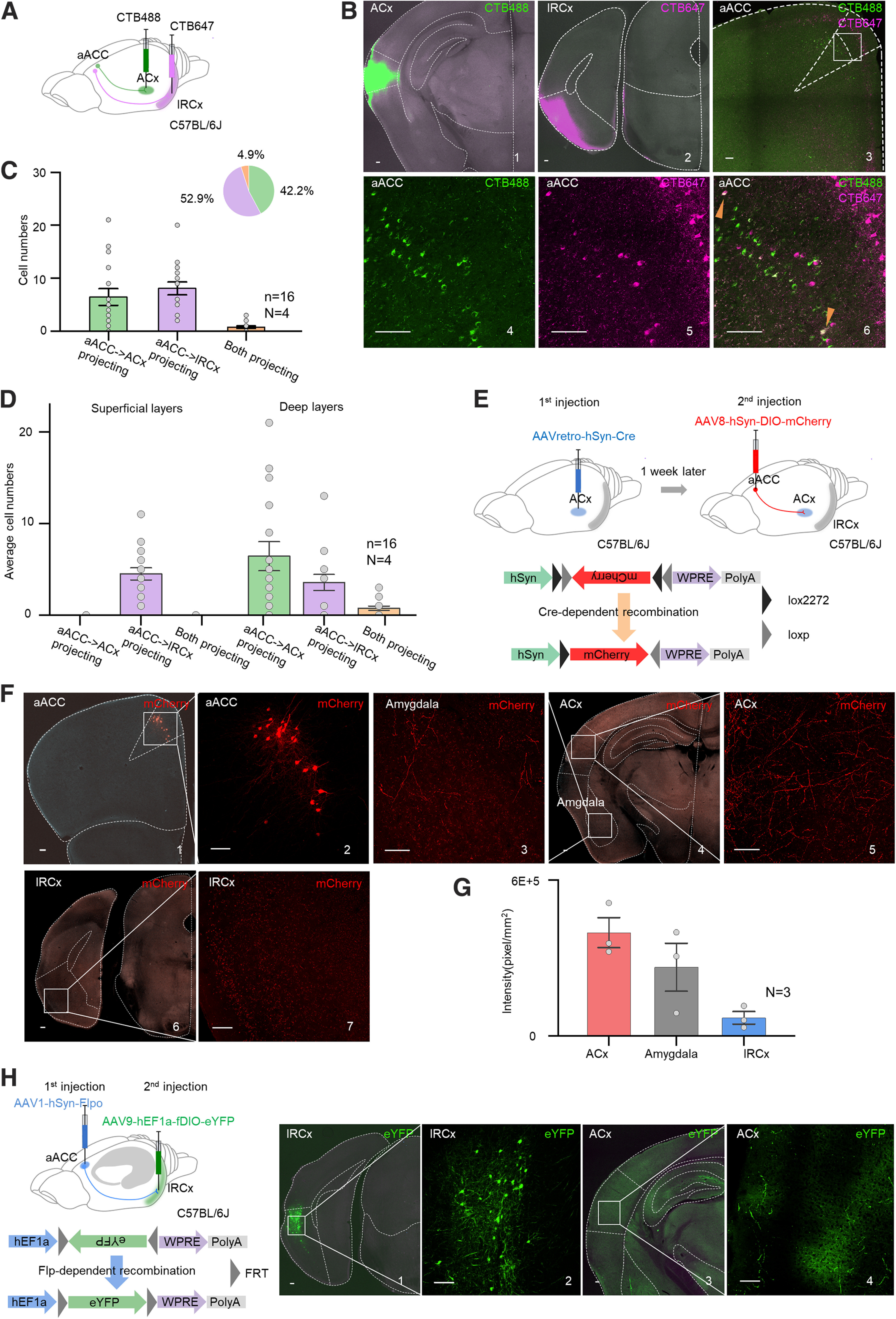
Two groups of ACC neurons separately projected to the ACx and lRCx. ***A***, Schematic drawing showing CTB647 injected into the lRCx and CTB488 injected into the ACx. ***B***, Representative image showing CTB-injected sites (1, CTB488 in the ACx; 2, CTB647 in the lRCx) and the aACC (3-6). Scale bars, 100 µm. Arrows indicate merged cells. ***C***, Average and individual numbers of neurons labeled by CTB488 (green), CTB647 (purple), and both (yellow) in the aACC. Inset, Percentage of each type of neuron. ***D***, Average and individual numbers of neurons labeled by CTB488 (green), CTB647 (purple), and both (yellow) in superficial and deep layers of the aACC. ***E***, Schematic diagram showing AAVretro-hSyn-Cre injected into the ACx and AAV8-hSyn-DIO-mCherry injected into the aACC. ***F***, Representative images showing mCherry expression in the aACC (1 and 2), amygdala (3 and 4), ACx (4 and 5), and RCx (6 and 7). Scale bars, 100 µm. ***G***, Fluorescence intensities of mCherry in the ACx, amygdala, and lRCx. ***H***, Left, Schematic diagram showing AAV1-hSyn-Flpo injected into the aACC and AAV9-hEF1a-fDIO-eYFP injected into the lRCx. Right, Representative images showing eYFP expression in the lRCx (1 and 2) and ACx (3 and 4). Scale bars, 100 µm. *n* represents the total number of images. *N* represents the number of animals recruited in the experiment. Error bars represent SEM.

To understand how the lRCx mediates this potentiating effect, we investigated the anatomic connections among the aACC, lRCx, and ACx. Distributions of retrogradely labeled neurons were examined after CTB488 and CTB647 were infused into the ACx and lRCx, respectively, in WT animals (Fig. 4*A*). Most labeled neurons in the aACC projected only to the ACx (42.2%, Fig. 4*B*,*C*, green signals and green bar) or to the lRCx (52.9%, Fig. 4*B*,*C*, purple signals and purple bar). ACx-projecting neurons concentrated in deep layers, whereas lRCx-projecting neurons were located in both superficial and deep layers (Fig. 4*D*). To further validate the separation of these two groups of neurons in the aACC, AAVretro-hSyn-Cre was injected into the ACx of WT mice followed by injection of a Cre-dependent virus (AAV8-hSyn-DIO-mCherry) into the aACC to label neurons projecting to the ACx (Fig. 4*E*). Similar to CTB488 labeling, neurons in deep layers of the ACC expressed mCherry (Fig. 4*F*). For the projections, high levels of mCherry signals were detected in the ACx and amygdala (Fig. 4*F*,*G*). By contrast, signals were sparse in the RCx (Fig. 4*F*,*G*). Therefore, two groups of neurons in the aACC appeared to separately govern the ACx and lRCx. The following question would be whether those RCx neurons receiving projections from the aACC send axons to the ACx. To answer this question, an anterograde trans-synaptic virus (AAV1-hSyn-Flpo-WPRE-pA) was injected into the aACC region of WT mice followed by injection of an Flp-dependent virus (AAV9-hEF1a-fDIO-eYFP-WPRE-pA) into the lRCx 1 week later to label lRCx neurons receiving projections from the ACC (Fig. 4*H*). Four weeks later, eYFP fluorescent signals were found in the ACx, representing direct projections from lRCx neurons innervated by the aACC. These findings demonstrate the existence of an indirect pathway from the aACC to the ACx via the lRCx in addition to a direct projection from the aACC to the ACx.

### Silencing the lRCx blocks the enhancing effect of the HFLaser protocol in the aACC

**Figure 5.**
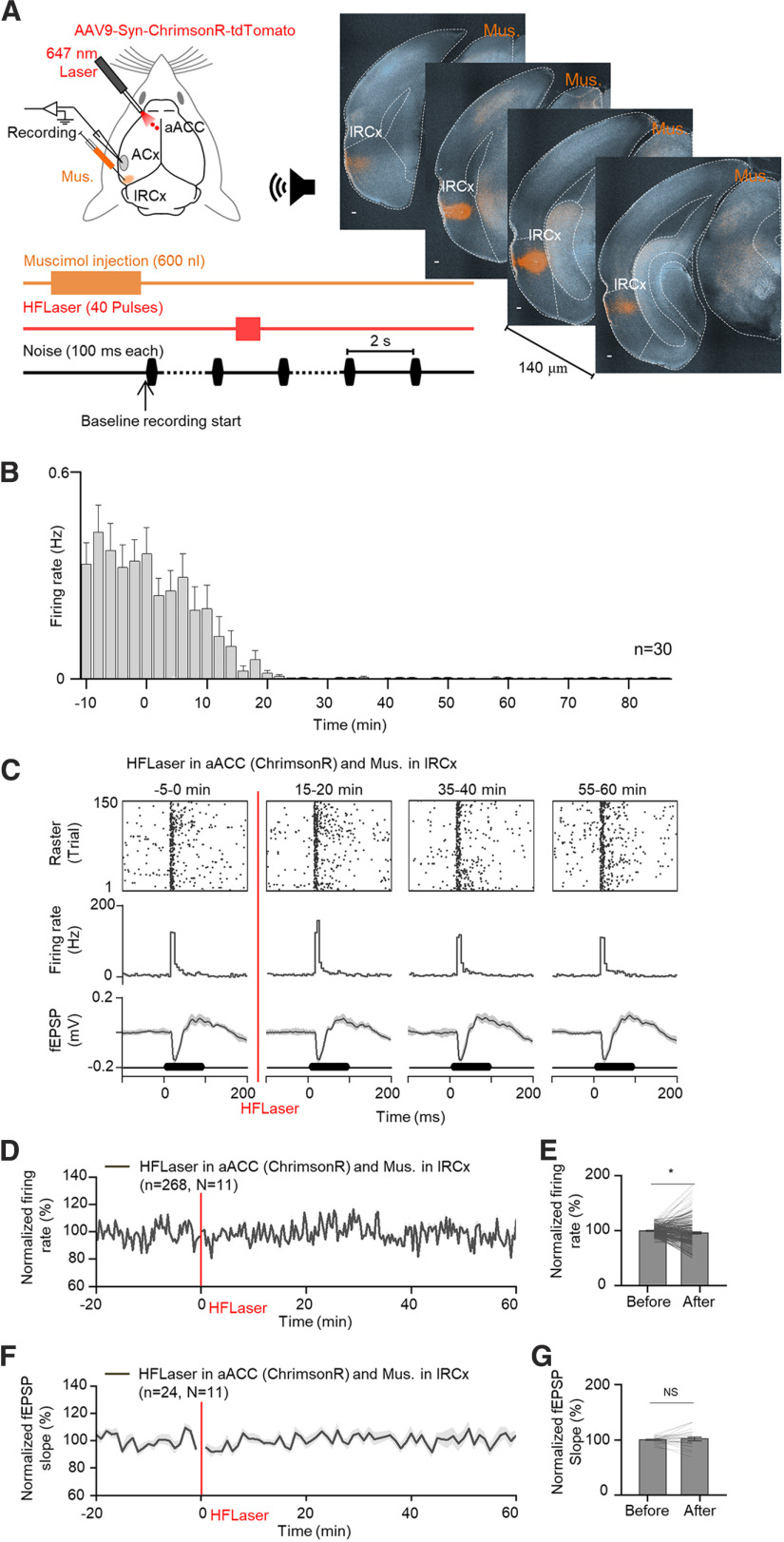
Silencing the lRCx eliminated the enhancing effect of the HFLaser protocol in the ACC. ***A***, Left, Schematic diagram showing the experimental setup and protocol. Right, A series of representative images showing the infusion area of muscimol in the lRCx. Scale bar, 100 µm. ***B***, Average spontaneous responses in the lRCx at different time points before and after the start of muscimol infusion. ***C***, Auditory responses from a representative recording site (top: raster plots; middle: PSTHs; bottom: fEPSPs) at different time points (−5 to 0, 15-20, 35-40, and 55-60 min) relative to the HFLaser protocol applied at the aACC in mice injected with AAV9-Syn-ChrimsonR-tdTomato in the ACC when muscimol was infused into the lRCx. ***D***, Average normalized firing rate tracings. ***E***, Average and individual normalized firing rates before (−10 to 0 min) and after (50-60 min) the HFLaser protocol. ***F***, Average normalized fEPSP slope tracings. ***G***, Average and individual normalized fEPSP slopes before (−10 to 0 min) and after (50-60 min) the HFLaser protocol. *n* represents the total number of recording sessions. *N* represents the number of animals recruited in the experiment. Shadows and error bars represent SEM. *p < 0.05; **p < 0.01; ***p < 0.001; NS, no significant changes; paired *t* test (for detailed statistics, see Table 1).

After showing the existence of this indirect pathway from the aACC to ACx via the lRCx anatomically, the next step was to examine whether the lRCx participates in the induction of long-term enhancement in the ACx by the HFLaser protocol in the aACC physiologically. Muscimol, a GABA A receptor agonist, was infused into the lRCx through a glass pipette (Fig. 5*A*). Muscimol infusion silenced neural activity in the target region for >1 h (Fig. 5*B*), which allowed us to test the effectiveness of the HFLaser protocol in the aACC while inhibiting neuronal activity in the lRCx. Immediately after muscimol injection (600 nl, 30 nl/min), neuronal responses of noise-evoked fEPSPs and MUAs in the ACx were recorded (Fig. 5*A*). Following a 20 min baseline recording, the HFLaser protocol was applied in the aACC, and responses in the ACx were monitored for another hour. After the lRCx was silenced, high-frequency activation of aACC neuronal cell bodies no longer enhanced auditory responses in the ACx for both auditory-evoked fEPSPs and MUAs (Fig. 5*C* for example recording site, Fig. 5*D*,*E*, 99.8 ± 0.5% vs 96.0 ± 1.6% for normalized firing rates of MUAs, and Fig. 5*F*,*G*, 99.8 ± 1.11% vs 101.6 ± 3.5% for normalized fEPSP slopes). This loss-of-function experiment demonstrates that an intact lRCx was indispensable for the ability of the aACC to facilitate auditory responses in the ACx long-term.

### High-frequency activation of aACC-projecting terminals in the lRCx induces long-term enhancement of auditory responses in the ACx

**Figure 6.**
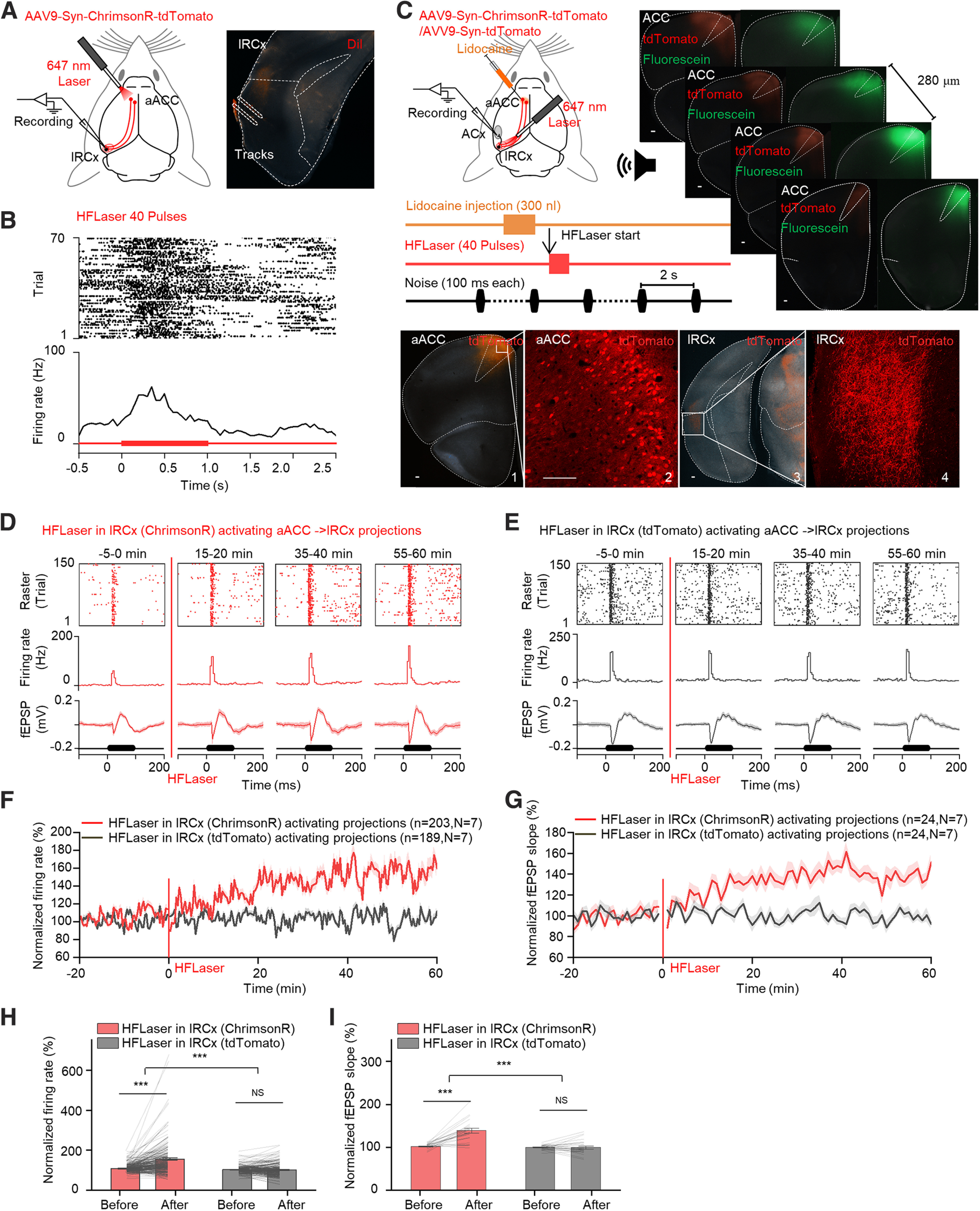
High-frequency activation of aACC-to-lRCx-projecting terminals induced long-term enhancement of auditory responses in the ACx. ***A***, Left, Schematic diagram showing the experimental setup. Right, Representative image showing the electrode tracks generated by one penetration of the array inserted into the lRCx. ***B***, HFLaser-evoked responses at a representative recording site in the lRCx (top: raster plot; middle: PSTH). ***C***, Top left, Schematic diagram showing the experimental setup and protocol. Top right, A series of representative images showing the infusion area of lidocaine in the aACC. Scale bar, 100 µm. Bottom, Representative images showing tdTomato expression in the injection site (1 and 2) and RCx (3 and 4). Scale bars, 100 µm. ***D***, Auditory responses from a representative recording site (top: raster plots; middle: PSTHs; bottom: fEPSPs) at different time points (−5 to 0, 15-20, 35-40, and 55-60 min) relative to the HFLaser protocol applied at the lRCx in mice injected with AAV9-Syn-ChrimsonR-tdTomato in the aACC (Condition 1). ***E***, Same as in ***D***, except for the HFLaser protocol was applied at the lRCx in mice injected with AAV9-Syn-tdTomato (control virus) in the aACC (Condition 2). ***F***, Average normalized firing rate tracings in Condition 1 (red curve) and Condition 2 (black curve). ***G***, Average normalized fEPSP slope tracings for conditions shown in ***F***. ***H***, Average and individual normalized firing rates before (−10 to 0 min) and after (50-60 min) the HFLaser protocol in Conditions 1 (red bar) and 2 (gray bar). ***I***, Average and individual normalized fEPSP slopes before (−10 to 0 min) and after (50-60 min) the HFLaser protocol for conditions shown in ***H***. *n* represents the total number of recording sessions. *N* represents the number of animals recruited in the experiment. Shadows and error bars represent SEM. *p < 0.05; **p < 0.01; ***p < 0.001; NS, no significant changes; two-way mixed ANOVA (for detailed statistics, see Table 1).

To further confirm the participation of the lRCx, we injected AAV9-Syn-ChrimsonR-tdTomato into the aACC region and inserted the electrode array into the lRCx while placing an optical fiber at the virus injection site (aACC, Fig. 6*A*). The HFLaser protocol evoked significant neuronal activity in the lRCx (Fig. 6*B*), showing the excitatory nature of the projection from the aACC to the lRCx. In the next experiment, the electrode array was inserted into the ACx to monitor auditory responses, and the optical fiber was moved to the lRCx to activate aACC-projecting terminals in the lRCx by the HFLaser protocol while cell bodies in the aACC were silenced by lidocaine (Fig. 6*C*). The same volume of fluorescein was administered to check the diffusion range of lidocaine, which overlapped well with the virus expression region in the aACC. The HFLaser protocol in the lRCx induced a similar long-term enhancement of auditory responses as aACC neuronal cell body excitation (Fig. 6*D* for example recording site, Fig. 6*F*,*G*, red curves, and Fig. 6*H*,*I*, red bars, 108.3 ± 1.5% vs 155.7 ± 6.1% for normalized firing rates of MUAs and 101.8 ± 1.2% vs 138.9 ± 5.4% for normalized fEPSP slopes), which supports the hypothesis that the aACC implements this enhancing effect through the lRCx. In mice in which a control virus (AAV9-Syn-tdTomato) was injected, no significant change was observed after the HFLaser protocol (Fig. 6*E* for example recording site, Fig. 6*F*,*G*, black curves, and Fig. 6*H*,*I*, gray bars, 102.7 ± 1.4% vs 101.6 ± 2.5% for normalized firing rates of MUAs and 99.6 ± 1.2% vs 99.4 ± 3.6% for normalized fEPSP slopes).

### High-frequency activation of aACC-projecting terminals in the lRCx facilitates sound-evoked flight responses long-term

**Figure 7.**
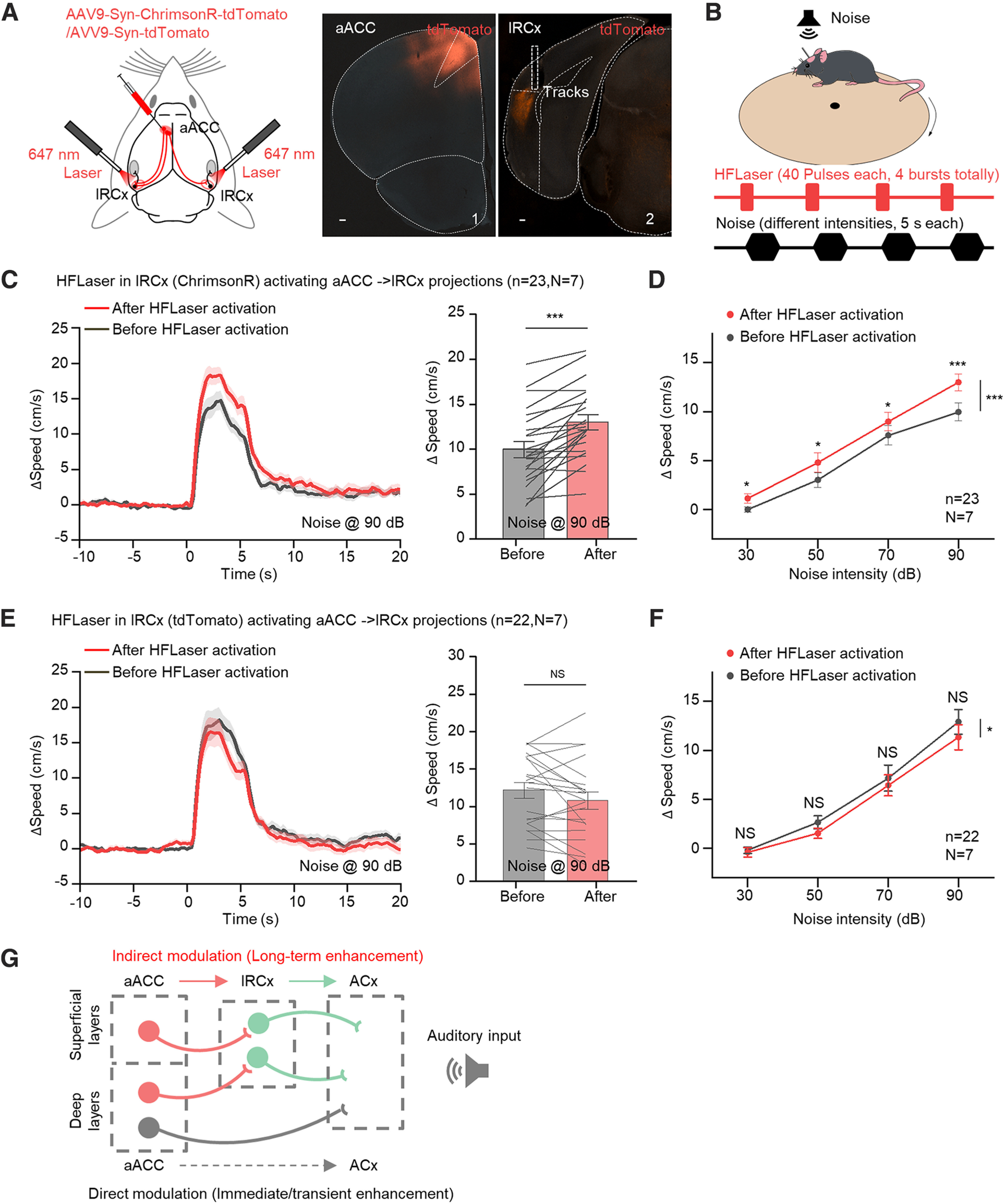
High-frequency activation of aACC-to-lRCx-projecting terminals facilitated sound-evoked flight responses long-term. ***A***, Left, Schematic drawing showing AAV9-Syn-ChrimsonR-tdTomato or AAV9-Syn-tdTomato injected into the aACC and optical fibers implanted in the lRCx. Right, Representative images showing tdTomato expression in the injection site (1) and RCx (2). Scale bars, 100 µm. ***B***, Schematic diagram showing the experimental setup and protocol. ***C***, Left, Representative speed change tracings to sounds at 90 dB (sound pressure level [SPL]) before (black) and after (red) the HFLaser protocol in the lRCx of mice injected with AAV9-Syn-ChrimsonR-tdTomato in the aACC. Right, Average and individual speed changes during the sound (90 dB)-presenting period (0-5 s) before and after the HFLaser protocol. ***D***, Average speed changes at different sound intensities (30, 50, 70, and 90 dB) before (black) and after (red) the HFLaser protocol in the lRCx of mice injected with AAV9-Syn-ChrimsonR-tdTomato in the aACC. ***E***, Left, Representative speed change tracings to sounds at 90 dB (sound pressure level [SPL]) before (black) and after (red) the HFLaser protocol in the lRCx of mice injected with AAV9-Syn-tdTomato in the aACC. Right, Average and individual speed changes during the sound (90 dB)-presenting period (0-5 s) before and after the HFLaser protocol. ***F***, Average speed changes at different sound intensities (30, 50, 70, and 90 dB) before (black) and after (red) the HFLaser protocol in the lRCx of mice injected with AAV9-Syn-tdTomato in the aACC. ***G***, Proposed direct and indirect pathways and their respective functions from the aACC to the ACx. *n* represents the total number of behavioral sessions. *N* represents the number of animals recruited in the experiment. Shadows and error bars represent SEM. *p < 0.05; **p < 0.01; ***p < 0.001; ***C***, ***E***, NS, no significant changes; paired *t* test; ***D***, ***F***, two-way repeated ANOVA (for detailed statistics, see Table 1).

Thus far, our study demonstrates that the aACC indirectly induced the long-term enhancement of auditory responses in the ACx via the lRCx anatomically and physiologically. Finally, we conducted a behavioral experiment in which we optogenetically activated aACC-projecting terminals in the lRCx and tested whether this manipulation could enhance sound-evoked flight responses long-term. AAV9-Syn-ChrimsonR-tdTomato was injected into the aACC region, and optical fibers were implanted into both sides of the lRCx (Fig. 7*A*). A sound-evoked flight paradigm was adopted to measure the flight response to noise (Fig. 7*B*). First, 5 s noises at 30, 50, 70, and 90 dB were presented, and animals' running speeds were measured as baseline responses. Next, four pairings of the HFLaser protocol and noise were applied to induce the presumed long-term enhancement of auditory responses in the ACx (Fig. 7*B*). One hour later, the same series of 5 s noises was presented to examine the long-term effect. As an example, for 90 dB, the HFLaser protocol applied in the lRCx significantly facilitated sound-evoked flight responses (Fig. 7*C*, 9.97 ± 0.89 vs 12.99 ± 0.83 cm/s). In mice injected with the control virus (AAV9-Syn-tdTomato), no effect was observed after the HFLaser protocol in the lRCx (Fig. 7*E*, 12.90 ± 1.23 vs 11.33 ± 1.24 cm/s). Group data show that the HFLaser protocol in the lRCx significantly elevated the speed-to-intensity psychometric curve in mice injected with AAV9-Syn-ChrimsonR-tdTomato (30 dB: 0.01 ± 0.26 vs 1.13 ± 0.46 cm/s; 50 dB: 3.03 ± 0.74 vs 4.80 ± 0.97 cm/s; 70 dB: 7.58 ± 0.97 vs 8.99 ± 0.92 cm/s; 90 dB: 9.97 ± 0.89 vs 12.99 ± 0.83 cm/s; Fig. 7*D*) but not in mice injected with the control virus (30 dB: −0.19 ± 0.31 vs −0.41 ± 0.45 cm/s; 50 dB: 2.65 ± 0.66 vs 1.54 ± 0.51 cm/s; 70 dB: 7.16 ± 1.30 vs 6.43 ± 1.05 cm/s; 90 dB: 12.90 ± 1.23 vs 11.33 ± 1.24 cm/s; Fig. 7*F*). Therefore, when aACC-to-lRCx projecting terminals were excited at high frequency, long-term enhancement of auditory responses was also induced behaviorally.

## Discussion

Previous study showed that the activation of ACC projections by a single pulse of laser in the ACx potentiated auditory responses transiently (Sun et al., 2022). Here, we demonstrate that aACC high-frequency activation could evoke LTP of auditory responses in the ACx. Instead of a direct projection from the aACC to the ACx, this effect was generated via an indirect pathway through the lRCx (Fig. 7*G*). Along with the short-term effect promoted by ACC direct projections, the survival odds of animals may be elevated, as animals can have speedier responses when facing ensuing threats after a significant event. The detailed operating mode of direct and indirect routes is still unknown. We suppose that the two pathways can be activated at the same time when mice face a crucial incident. However, their engagement under usual circumstances may depend on the level of significance. With low to moderate levels, only the direct pathway is activated, and the high level would also energize the indirect pathways.

Electrophysiological experiments were conducted on both adult male and female C57BL/6J mice, and we did not record the sex of the animals in this part of the study. By blocking neuronal somas in the ACC with lidocaine, we showed that the long-term potentiating effect in the ACx was not caused by a change in the ACC itself when stimulating the projections from the ACC to the lRCx (Fig. 6*C-I*). Nevertheless, we cannot rule out the possibility that the long-term change occurred in the lRCx, which drove the change in the ACx, although the brief recording in the lRCx did not show this tendency (Fig. 6*A*,*B*). The same issue pertains to when we directly stimulated aACC neuronal somas to induce a long-lasting enhancement in the ACx (Fig. 2). The change could arise in the ACC, the ACx, or both. A future experiment in which both lRCx-to-ACx and ACC-to-lRCx pathways are specifically inhibited after the ACC stimulation would be necessary. The frequency range of the acoustic noise stimuli adopted in this study is limited at 12.5 kHz because of the 25 kHz sampling rate, which is relatively low compared with the mouse hearing range (2-3 kHz to 69-90 Hz). Acoustic noise stimulus at higher frequencies will be examined in future studies focusing on the specificity of the long-term enhancement.

In our study, ACC neurons were activated by the laser stimulation. The natural condition that can induce such substantial activity in the ACC and then activate the indirect pathway awaits future exploration. The air puffing used in our previous study at a higher intensity may be a good start. Other choices include a flashlight, looming visual stimuli (Shang et al., 2018), or predator odors (Ferrero et al., 2011). Further studies could then examine how the aACC-to-lRCx-to-ACx pathway is activated by such stimuli with high valence.

Numerous studies have investigated how higher-order cortices modulate activity in sensory areas. Through direct projections, higher-order cortices can dramatically change the responsiveness of neurons in the sensory cortex by mechanisms, such as synchronizing oscillations in different frequency bands (Helfrich et al., 2017) or regulating the inhibitory system (Nelson et al., 2013). On the other hand, higher-order cortices also exert influence through indirect projections via the thalamus (Giraldo-Chica et al., 2018; Yang et al., 2022) or basal ganglion (Nakajima et al., 2019). Few studies have focused on indirect modulation through a cortical region (Conner et al., 2010), especially its long-term effect. Here, we showed how a higher-order cortex induces long-term change by activating another cortical area apart from an immediate and transient effect. The ACC and RCx also show intensive innervation to other modalities (e.g., visual cortex) (S. Zhang et al., 2014; Sidorov et al., 2020; Lu et al., 2022). It is worth examining these pathways and investigating whether they bear similar properties.

Our previous study shows that the entorhinal cortex promotes the formation of LTP and associative memory in the ACx through its cholecystokinin-positive projections (X. Chen et al., 2019; Z. Zhang et al., 2020). We presume that the long-term enhancement observed in this study is induced by the same mechanism, although we cannot rule out other possibilities, including other neuromodulators (e.g., acetylcholine) (Ma and Suga, 2005; Guo et al., 2019) and plasticity of the inhibitory system (S. Zhang et al., 2014; Topolnik and Tamboli, 2022). As to the nature of the long-term enhancement, we consider that it may be a specific memory trace (similar to the memory trace formed in fear conditioning) (Isoardi et al., 2004; Kim and Cho, 2017; de Lima et al., 2022), although we did not measure its specificity by presenting different auditory stimuli. The ACx drives innate sound-induced flight behavior through its projection to the inferior colliculus, which controls the dorsal periaqueductal gray to initiate the flight response (Xiong et al., 2015; H. Wang et al., 2019). As an upstream area of ACx, the ACC commands various other downstream areas in addition to the lRCx, such as the thalamus (S. Zhang et al., 2016), superior colliculus (Huda et al., 2020), amygdala (Jhang et al., 2018), and zona incerta (Chou et al., 2018). These areas could also participate in the modulation of flight behavior by interacting with the ACx, inferior colliculus, and periaqueductal gray. How these areas cooperate to generate appropriate flight behaviors coherently is pending for future studies.

The relationship between the ACC's sensory enhancing capacity and its other functions could be complex and multifaceted. On one hand, the ACC's ability to enhance sensory perception can impact its other functions (e.g., cognitive control). For instance, the ACC is involved in directing attention toward important sensory stimuli (S. Zhang et al., 2014). Enhanced sensory perception can improve an individual's capability to detect important sensory cues, allowing the ACC to more effectively direct attention toward these cues (Sun et al., 2022). Sensory information is also a critical component of emotional experiences. Enhanced sensory perception can therefore impact emotional responses generated by the ACC (Etkin et al., 2011; Jhang et al., 2018). On the other hand, the ACC's other functions (e.g., integrating negative affect, cognitive control, reward processing) can also impact its ability to enhance sensory perception (Shackman et al., 2011; T. Chen et al., 2014). For example, the ACC engages in cognitive control processes, such as error monitoring and conflict resolution (Carter et al., 1998; Wu et al., 2017). These processes can shape the ACC's ability to allocate attention and resources toward sensory processing, which can in turn modify sensory perception. The ACC is also involved in social cognitive processes, such as empathy and perspective-taking (Hughes and Beer, 2012; Basile et al., 2020). These processes can impact sensory perception by influencing how individuals attend to and interpret social cues. Therefore, the ACC's sensory modulating capacity tightly integrates with its other higher-order functions, optimizing its operation.

As the effect generated by this indirect aACC-to-ACx projection can last for a long time, it would intensify the overexcitement of the auditory system triggered by the direct projection from the ACC under pathologic conditions. The combined effect would prolong and exaggerate symptoms related to the overexcited system. Further studies showing how this pathway contributes to the development of related brain disorders would help increase our understanding of pathogenesis and the design of new treatments.
